# Surgical Club of the South West

**Published:** 1987-02

**Authors:** 


### Surgical Club of South West England

### Meeting at Plymouth May 1986
### Combined with the Council of the Royal College of Surgeons of England

### GASTRO-DUODENAL CROHN'S DISEASE

**P. R. Maddox**
**Plymouth**

A case of gastro-duodenal Crohn's disease was presented, in whom there was also intestinal and colonic involvement. He subsequently underwent a partial gastrectomy with Roux-en-Y anastomosis, dissecting the first 18 ins of jejunum, which was macroscopically involved with Crohn's disease and post-operatively fared very well. As this was an unusual case and gastro-duodenal Crohn's disease is relatively rare, this lead me to review the literature of gastro-duodenal Crohn's disease.

Crohn's disease is a truly pan-intestinal disease and can affect any part of the alimentary tract from the mouth to the anus. Gastro-duodenal Crohn's was first described by Ross (1) in 1949 and has a documented prevalence of 0.5–7.0% (2) in patients with Crohn's disease, although recent evidence may suggest it is more common (approximately 22–24%). (3) Over the past 37 years only about 200 cases have been reported and the optimum treatment for Crohn's disease affecting the stomach and/or duodenum is uncertain. Medical therapy is generally accepted for non-obstructive disease but gastro-duodenal Crohn's disease often progresses to stenosis with associated gastric outlet obstruction, requiring either by-pass surgery or resection (4). There are protagonists for all modes of treatment, but as has been learned with intestinal Crohn's, a more conservative approach is to be preferred if at all possible (5). Stricture-plasty and even endoscopic balloon dilatation have recently been advocated (6). In particular I think the medical treatment with steroids, Sulphasalazine and total parenteral nutrition, although not the panacea for all patients, is indicated before surgery is considered, and certainly warrants further study.

#### REFERENCES

1. ROSS, 1949 R. Cicatrising Enteritis, Colitis and Gastritis. Gastro-Enterotology 1949, **13**; 344–350.
2. PRIEBE, W., SMITH, J. B. 1983 Crohn's Disease of the Stomach with Outlet Obstruction colon. A Case Report and Review of Therapy. J. Clin. Gastroenterol. 5 Colon; 441–445.
3. KORELITZ, B. I., et al. 1981 Colon. Crohn's disease in endoscopic biopsies of the gastric antrum and duodenum. An. J. Gastroenterol. 76 Colon; 103–109.
4. KYLE J. 1982 Gastro-duodenal involvement in Crohn's Disease. J. Roy. Coll. Surg. Edin. 27 (6); 327–332.
5. ALEXANDER-WILLIAMS J. 1976 Crohn's Disease and the Surgeon. Crohn's Surgical Practice. Volume 1. R.C.S.E. 192–202.
6. ALEXANDER-WILLIAMS J. Surgery for Crohn's Disease. Surgical Abstracts. A review of current topics in Surgery. Vol. II, No. 1. (Lederle tapes.)

### THE BLOOD FLOW OF THE OBSTRUCTED COLON

**J. E. Coxon**
**Plymouth**

Acute intestinal distension has been shown experimentally to impair local blood flow and ischaemia is a known factor in anastomotic dehiscense. However the haemodymanic consequences of a chronic obstruction, such as that resulting from a progressive colonic cancer are unknown. A model of chronic progressive large bowel obstruction was developed in the mini-pig. This mimics the mechanical component of carcinomatous large bowel obstruction. Blood flow before and after obstruction of the upper rectum was measured by two techniques; the local injection of Xenon$^{133}$ radioisotope and the intracardiac injection of radiolabelled microspheres.

The blood flow of the left colon, proximal to the obstruction, showed a 5 fold increase and the ileal blood flow doubled. In contrast there was a 13% fall in the blood flow to the caecum. Differential studies of mucosal and muscle blood flows showed a shunting of blood from the mucosa to the muscle and this shunting was most marked in the caecum.

These results may explain why the caecum is the first site to become gangrenous or perforate even when the obstruction is in the upper rectum. The study provides no contraindication to the primary anastomosis of the obstructed left colon.

### EXPERIENCE WITH THE POUCH

**W. H. F. Thomson**
**Gloucester**

Restorative proctocolectomy is in its infancy and different techniques are offered by experts both in the mode and amount of rectal resection, the type of pouch construction, and the method of its suture and endo-anal anastomosis.

I have used the W Pouch – with a single layer extra-mucosal edge-to-edge apposition for optimal constructional capacity – on a consecutive series of 15 patients in the last two years, 14 for ulcerative colitis and one for polyposis coli. Three await closure of the defunctioning ileostomy.

One patient suffered a pelvic haematoma which became infected after presumably incomplete evacuation and required re-drainage. There have been no other problems, and no failure of Pouch construction of anastomosis.

The functional result in 11 of the 12 completed cases is encouraging. Six have two to three bowel actions a day; the others between four and six. Only one, who already had post-vagotomy diarrhoea takes gut sedatives. None have urgency. Mucus seapage occurs in most (though not all) but is only very slight and mainly at night. All have returned to full normal activities.

Functional dissatisfaction afflicts one patient, the only polyposis sufferer in the group. Chronically depressed (on drugs) and of inadequate personality, she was, in retrospect, unsuited for the procedure. The rest are delighted with their result and one has become pregnant. Only time will establish the operation's durability, however.

### OESOPHAGECTOMY WITHOUT THORACOTOMY

**Professor R C N Williamson**
**University Department of Surgery**
**Bristol Royal Infirmary**

Between September 1981–April 1986 in this unit 12 patients have undergone abdominocervical oesophagectomy, ie subtotal excision of the oesophagus by a transhiatal approach without the need for a formal thoracotomy but with anastomosis in the neck. Of 10 patients with carcinoma, 9 have been over the age of 70 years (including 2 octogenarians). Two patients developed recurrent achalasia and megaoesophagus 30 years after Heller's cardiomyotomy. There have been no anastomotic leaks and operative deaths, though chest complications were common. Four patients had a wide posterior mediastinum (achalasia 2, rolling hiatal hernia 2), and this facilitated the 'blind' dissection from below. Four patients have died of recurrent carcinoma at 4, 12, 17 and 21 months, but the other 8 are alive (maximum 20 months). Benign anastomotic stricture has required bougienage in 4 cases. This operation may be a reasonable palliative option in elderly patients who might not survive a full thoracolaparotomy.

### COMPARISON OF INTERCAVITY IRRADIATION PULSION INTUBATION AND TRACTION INTUBATION FOR CARCINOMA OF OESOPHAGUS

**K. M. Pagliero**
**Exeter**

With the advent of after loading techniques we have been able to develop an applicator, that can be accurately sited endoscopically and with radiological control within an oesophageal cancer. Using the Selectron (Nucletron, Holland) we have been able to treat lesions with 48 Caesium 137 sources to a dose of 1500 cGy at 10 mm off central axis as a 12 cm line source over a period of 1.14 hours. In appropriate cases the entire oesophagus can be irradiated in two consecutive applications.

In our pilot study 72 patients deemed inoperable either due to unresectable disease or turned down for major surgery on fitness grounds, underwent brachytherapy. Sixty-nine tolerated the treatment of which 80% had useful improvement in swallowing. One patient with trachial involvement developed a fistula and would not now be so treated. One patient developed radiation stricture which responded to bougienage. Squamous cell carcinoma and adenocarcinoma responded equally favourably.

Compared to our experience with traction (TI) and pulsion (PI) intubation there were distinct advantages with brachytherapy (B). Mortality was zero with selectron (T.I. 23% P.I. 7%). Hospital stay was reduced (B 3 days; T.I. 17 days; P.I. 14 days) overall completion rates were reduced (B 10%; T.I. 38%; P.I. 43%) and survival at 6 months was increased (B25%; T.I. 15%; P.I. 5%). Brachy therapy is thus our first line palliative treatment for inoperable oesophageal carcinoma.

### *CARCINOMA OF THE OESOPHAGUS. IS RESECTION WORTHWHILE?*

**B. Pickering and J. Rahamin**
**Derriford Hospital, Plymouth**

The treatment offered for oesophageal cancer should aim firstly to restore the ability to swallow and secondly—hopefully—to prolong the patient's survival. Surgical resection is still currently the only treatment to offer these, but it must be associated with a low mortality rate if it is to remain the first choice in all cases.

From this relatively new Unit the first 100 resections for oesophageal cancer have been analysed.

58 were male, 42 female with an age range from thirty to eighty; 30 patients were over 70 years; 58% adenocarcinoma, 42% squamous and there was macroscopic coeliac gland involvement in 47% of cases. Duration of symptoms varied widely from 2 weeks to 18 months. There were no deaths at operation. There were six hospital deaths, 4 from cardiovascular accidents and 2 from leaks. 34 patients are alive and in their 2nd to 7th year after operation. The survival times of the remainder varied between 2 and 56 months, all eating and swallowing normal food.

Even witha 6% hospital mortality we feel that resection wherever possible is worthwhile.

### WHITHER TRANSPLANTATION

**R. Y. Calne**
**Cambridge**

When a donor liver has been obtained 10 hours is the maximum delay before it can be successfully transplanted into a patient and helicopters and police motor cycle teams may be needed for its transport. No account of tissue typing is taken as this would further prolong the delay. The most difficult part of the operation is often the removal of the diseased liver, particularly if there has been previous surgery. Sometimes it is advisable to assist the circulation with a bypass, taking blood from the IVC to the aorta without using heparin as this produces severe bleeding problems. The advent of Cyclosporin A has greatly simplified the problems of immune suppression. The postoperative care is so incredibly complex that there has to be a 1;1 doctor/patient ratio in the ICU. Over *300* liver transplants have now been done with survival up to *11* years. One third of the grafts are done in children and there is a better than 90% graft survival, though some of them have needed a second transplant. Pancreas transplantation is best indicated when a diabetic patient with renal failure is in danger of loosing his sight from retinitis. The tail of the pancreas is transplanted and the duct brought into the stomach, the vascular connections are made with the splenic vessels with a self closing a-v shunt to assist patency. A renal transplant is done at the same time. The early results are encouraging.

M. G. W. for R. Y. C.

### "STONE-BASHING ON DARTMOOR"

**J. C. Hammonds**
**Plymouth**

The management of upper urinary tract stones has radically changed in the past five years. Ureterorenoscopy, percutaneous nephrolithotripsy (P.C.N.), electrohydraulic lithotripsy (E.H.L.) and ultrasonic lithotripsy (U.S.L.) have been available in Plymouth since the beginning of 1985.

The results of treatment of upper urinary tract stones in the Plymouth Health District during the twelve months since the introduction of these techniques is presented.

Thirty patients underwent thirty-one P.C.N. procedures. There were twenty-six successful stone removals. In eighteen this was performed as a one stage procedure. Four patients had a previous tract established for emergency decompression of acute obstruction. Fourteen stones were removed intact, the remainder were disintegrated with E.H.L. or U.S.L. Over fifty per cent of the patients were discharged within seventy-two hours of the procedure.

Twenty-seven patients underwent twenty-nine ureteroscopic stone removals. Successful stone visualisation and disruption was achieved in twenty-three. The success rate was higher for stones in the lower third of the ureter. Most patients were discharged within fourty-eight hours of the procedure. These techniques have significantly altered the pattern of stone surgery in this district in that only a single open surgical exploration for stone was necessary in twelve months and a significantly increased number of patients have had their stones removed.

### PREDISPOSING FACTORS IN THE TUR REACTION

**M. N. Goble**
**Plymouth**

The transurethral resection reaction is a symptom complex resulting from the intraoperative absorption of irrigating fluid, and the ensuing dilutional hyponatraemia, occurring during transurethral prostatectomy. This symptom complex is synonymous with that which occurs in association with the syndrome of inappropriate antidiuretic hormone secretion ie. water intoxication secondary to compulsive water drinking. It is apposite to compare these two syndrome as I hope to demonstrate that serum antidiuretic hormone levels are also implicated in the transurethral resection reaction (TURR).

It has long been established that operative techniques are important in the aetiology of the TURR, and include: (a), operative time; (b), pressure of the irrigant fluid, and; (c), the number of opened prostatic capsular veins. However, the majority of patients undergoing transurethral prostatectomy have demonstrable evidence of irrigant absorption yet less than 10% of patients suffer the TURR. Hypothetically, therefore, it appears possible that there is a further aetiological factor involved such that some patients are able to tolerate a fluid load whereas others are not. To validate this theory, intraoperative changes in serum sodium concentration were correlated with the preoperative urine osmolality (as an indicator of antidiuretic hormone (ADH) status). Those patients with urinary osmolalities above the mean (480mosm) were found to be at greater risk of developing dilutional falls in serum sodium concentration ($p<0.02$, chi squared four-fold table with Yates modification). This result suggested that ADH may be responsible for this apparent variation in the fluid handling capabilities of patients undergoing prostectomy. As further confirmation, intraoperative serum ADH levels are now being measured; preliminary results show a correlation of peak operative serum ADH with the degree of dilutional hyponatraemia.

### PSYCHOLOGICAL ASPECTS OF SURGERY FOR ABNORMAL APPEARANCE

**David Harris**
**Plymouth**

Plastic surgeons lack a scientific basis by which to judge pre- and post-operatively the psychotherapeutic value of treating patients with abnormalities of appearance. An anecdotal survey of 54 such patients has revealed consistency in the symptomatology of abnormal appearance across a broad range of disfigurements. These symptoms have been sorted into six sub-sets which reflect a theoretical understanding of the psychological reactions which caused them:

(1) Those relating to the induction and development of self-consciousness: critical attitudes of others, self-comparison with others normal, mistaken identity;
(2) Those relating to a defence mechanism which the subject develops both to hide the abnormality from the sight of others and to disguise self-consciousness of it: camouflage techniques, restricted life style, artificial behaviour:
(3) Those relating to the experience of unavoidable distressing activities, e.g. hostile teasing at school;
(4) Those relating to a downgrading of the subject's self-concept, inferiority, etc.;
(5) Those relating to consequent difficulties with interpersonal relationships;
(6) Those relating to unsuccessful attempts to rationalise. Each of these symptoms causes the patient psychological distress and disables him/her from enjoying a normal lifestyle. Their identification preoperatively enables the surgeon to judge the potential psychotherapeutic benefits of operations designed to normalise appearance and the degree of their elimination post-operatively provides a measure of the success of such operations.

### KELOIDS

**D. J. Hanley**

**Plymouth**

This word is widely misused to refer to any red, thickened, pruritic scar.

Scars becomes thickened in their early maturation due to an excess of collagen synthesis over collagen degredation, but over a period of months flatten and soften. However, if healing of the initial wound is delayed, e.g. by infection, ischaemia or tension, an hypertrophic scar results, thicker than normal. This also settles to a normal scar but is usually more protracted.

The hypertrophic scar eventually matures and remains confined to the actual area of the wound, whereas the keloid persists and invades the adjacent dermis. It is uncommon. It is identical histologically with an hypertrophic scar and is usually diagnosed retrospectively.

Keloids have an undoubted constitutional basis being common in Negroes, females and the young.

Certain anatomical sites such as the deltoid and presternal region have a high susceptibility.

Treatment is disappointing and should be aimed at prevention, by proper surgical technique, correct orientation of scars, and the avoidance where possible, of keloid-prone sites. For this reason, BCG vaccination in the deltoid should be condemned.

Pressure garments, intralesional triamcinolone and superficial radiotherapy are usually unhelpful in established keloids.

Excision alone results in recurrence and should always be combined with one of the above.

### CULTURED SKIN IN THE MANAGEMENT OF AN EXTENSIVE BURN

**C. Chapman**
**Plymouth**

The mortality of adult patients with extensive burns (defined here as burns involving more than 70% of body surface area) remains high in the United Kingdom.

It is hoped that now cultured skin is avilable the mortality in these casualties will be reduced.

M.C. a male patient aged 42 was admitted to the Burns Unit Derriford Hospital Plymouth three hours after sustaining scalds from a burst steam pipe. The body surface area involved was calculated at 78%.

After resuscitation the patient was taken to the operating theatre where an area of unburned skin in his left deltoid area was cleaned with alcohol. Threesplit skin grafts each approx 4cm×3cm were taken, wrapped in saline—gauze and placed in a Universal container which in turn was packed with ice. (Alternatively a full thickness elipse of skin could have been taken from his axilla). The specimen was taken to the skin culture Laboratory Birmingham Accident Hospital arriving there as requested less than three hours after it had been harvested.

In the laboratory the skin grafts were washed in saline, chopped into very small pieces with iris scissors and then digested with trypsin in a conical flask in an incubator for approximately thirty minutes. This digestion process was repeated six times. A cell count of the suspension was made and approximately two million liberated epithelial cells transferred to each culture flask. After ten days sub cultures were carried out, skin eventually being produced in forty culture flasks. The cultured skin was ready to transfer to the patient approximately three weeks after harvesting the skin grafts from the patient.

A week prior to the cultured skin being ready for transfer to Derriford the patients burns were debrided in the Operating theatre so that a granulating base was ready for the cultured skin when it was transferred.

The forty flasks containing cultured skin were transferred from the Birmingham Laboratory to Deriford Hospital by police car and on arrival were placed in an incubator at 37°C and in an atmosphere of 8% carbon dioxide.

The roofs of the plastic culture flasks were removed using a hot soldering iron, the culture fluid removed from the flasks and the enzyme dispase added. The Dispase helped lift off' the sheets of thin cultured skin from the underlying base so that the skin in each flask could be stapled to a piece of closely woven petroleum gauze without difficulty, so facilitating its movement to a petri dish pending its transfer to the granulating area on the patient. A routine skin graft dressing was then applied.

Other granulating areas on the patient were covered with conventional skin grafts which had been meshed.

There was a good take of the cultured skin. After further skin grafting the patient was discharged from hospital five months after his admission.

The cost of producing cultured skin compares favorably to treating these patients with porcine skin.

### STREPTOKINASE THERAPY FOR ACUTE ARTERIAL OCCLUSION

**B. P. Bliss**
**Plymouth**

There is renewed interest in thrombolytic therapy for acute arterial insufficiency. The drug is often delivered via indwelling arterial catheter but there may still be a case for simple intravenous therapy. In this series of 14 patients streptokinase was given by continous intravenous infusion. A loading dose of 750,000 units was followed by 250,000 units intravenously for 72 hours, followed by heparin 30,000 units per day and long term warfarin in successful cases. Selection for treatment involved the medically unfit (3), in-operability (6) and late diagnosis (5). All patients had limb threatening ischaemia, due to failed arterial reconstruction (6), primary arterial thrombosis (4) or late arterial embolis (4). Hyperpyrexia occurred in two patients but did not prevent completion of treatment. Treatment was discontinued in one patient because of purpura. One 90 year old patient died 17 days after successful thrombolytic therapy from ruptured abdominal aortic aneurysm. Successful recanalisation was achieved in six patients (three failed reconstructions, two late emboli, and one primary thrombosis). Of the eight failures, three patients claimed subjective improvement and only two went on to major amputation.

Thrombolytic therapy by intravenous infusion still has a place in the management of acute ischaemia for the unfit or in-operable patient.

### CAN SCROTAL ULTRASONOGRAPHY USEFULLY COMPLIMENT CLINICAL ASSESSMENT IN PREDICTING AN ADVERSE OUTCOME FOLLOWING EPIDIDYMO-ORCHITIS?

**K. M. Desai*, J. C. Gingell, J. M. Haworth**
**Departments of Urology and Radiology, Southmead Hospital, Bristol**

Epididymitis whilst usually resolving with appropriate antibiotic therapy may sometimes be complicated by orchitis, intrascrotal abscess, testicular infarction and atrophy. These may result from testicular ischaemia caused by extrinsic compression of its blood supply by constrictive funiculitis or by gross epididymal oedema. Under these circumstances prompt decompression by epididymotomy and lysis of the external spermatic fascia may prevent serious sequelae but selection for this procedure requires early identification of those testes at risk. We have attempted to do this by prospectively studying 31 men (age range 15–87 years) presenting to our Urology department with epididymitis. Besides various clinical parameters we also assessed the prognostic value of certain features on scrotal ultrasonography, in particular the epididymal and testicular size including the echo pattern of the latter and also the presence of a reactive hydrocele.

Testicular complications occurred in 14 men (45%). Severe inflammation with involvement of the cord, the presence of a co-existent bacterial UTI and the finding during the acute episode of an enlarged testis displaying reduced echogenicity when compared to the contralateral side were findings that were significantly associated with an adverse outcome.

Epididymitis seen in hospital practice often involves the testis, sometimes with serious consequences. Scrotal ultrasonography is a potentialiy valuable adjunct in its management and warrants further study to determine its precise role.

*(Paper omitted from report of previous meeting in Bristol, Oct 1985 and awarded to S.W. Surgical Prize.)

